# Cohort Profile: The Community Empowerment and Care for Well-being and Healthy Longevity Study

**DOI:** 10.2188/jea.JE20250357

**Published:** 2026-09-05

**Authors:** Akihiro Kakuda, Yuko Sawada, Rika Okumura, Tokie Anme

**Affiliations:** 1 Department of Physical Therapy, Morinomiya University of Medical Sciences, Osaka, Japan; 2 Graduate School of Health Science, Morinomiya University of Medical Sciences, Osaka, Japan; 3 Department of Public Welfare, Tobishima, Aichi, Japan; 4 Faculty of Medicine, University of Tsukuba, Tsukuba, Japan

**Keywords:** multi-generational cohort, life course perspective, community empowerment, healthy longevity

## Abstract

The Community Empowerment and Care for Well-being and Healthy Longevity (CEC) Study is an ongoing, multigenerational, population-based cohort designed to evaluate community-driven lifespan developmental care in a rapidly aging society. Conducted in Village T, Aichi Prefecture, Japan, whose demographic structure broadly resembles national patterns, the study includes residents from infancy to older adulthood. Since 2017, the CEC Study has maintained three age-specific sub-cohorts (0–19, 20–64, and ≥65 years), integrating questionnaire surveys, health examinations, home visits, and administrative records from medical, health, and long-term care systems. A total of 4,638 residents participated in the baseline survey, with 4,079 subjects responding. The study adopts a community empowerment approach, in which residents serve as co-designers and co-implementers of local health initiatives through workshops, monitoring activities, and public feedback sessions. Triennial follow-up surveys and continuous administrative data linkage enable long-term tracking of health trajectories and contextual influences across the life course. The dataset provides detailed information on physical and cognitive function, mental health, lifestyle, social participation, and environmental factors. Although generalizability may be greatest for regions with demographic and socioeconomic profiles similar to Village T, the CEC Study offers a valuable platform for evaluating real-world, community-led health strategies. Its integration of longitudinal data with empowerment-oriented practice provides insights relevant to evidence-based community health policy and global healthy aging initiatives.

## PURPOSE

The issue of rapid aging in modern society is not merely a challenge for older adults, but a societal obstacle that affects the well-being of all generations. Rapid demographic shifts and structural transitions across the life course have led to multiple emerging challenges, including widening intergenerational disparities, social isolation, declining labor force participation, and increasing caregiving and healthcare burdens among working-age populations.^1^^–^^5^ These intertwined issues highlight the importance of adopting a life-course approach to understand how demographic change impacts physical, mental, and social well-being throughout the lifespan. According to the United Nations,^6^ by 2060, the global population aged ≥65 years will reach 1.8 billion, with the rate of aging in Japan expected to rise to 28.2%. Such rapid demographic shifts and accompanying social structural changes impact all life stages from infancy to adulthood and are closely associated with social and economic factors.

Given these changes, the role of a life-course approach^7^^–^^10^ is increasingly emphasized in public health, considering the continuity and interactions of well-being across all age groups, rather than focusing on a particular age group. We define a life-course approach as the analysis of how risk and protective factors accumulate and interact from early life to old age, shaping later health and function. In CEC, this perspective is essential to quantify intergenerational linkages and the long-term benefits of early and mid-life interventions for healthy longevity. The World Report on Ageing and Health (2015)^11^ by the World Health Organization (WHO) also underscores the necessity of incorporating a life-course perspective in health policies, indicating that traditional studies involving specific age groups fail to comprehensively address societal health issues. Thus, a cohort study encompassing all age groups is essential to understand the intergenerational interactions between health and well-being.

Against this background of rapid demographic shift and intergenerational linkages, the lifespan developmental perspective^12^ has garnered attention. Lifespan developmental care involves implementing appropriate interventions for each life stage, from infancy to old age, with the potential to maximize individual well-being. Early interventions in youth and middle age contribute to improved self-management skills and development of healthy habits, significantly reducing the risk of chronic diseases and the need for long-term care in later years.^13^ Despite abundant evidence on effective health promotion and disease prevention at the individual level, there remains a critical gap in translating such evidence into population-level benefits through municipality-wide implementation. In particular, few longitudinal studies have quantified how resident participation and policy–program coordination are sustained over time and how these implementation processes relate to changes in health, healthcare use, long-term care, and costs. Addressing this gap requires a life-course, all-age cohort embedded in routine municipal operations and capable of tracking program reach, retention, fidelity, and iterative improvements. The Community Empowerment and Care for Well-being and Healthy Longevity (CEC) Study (https://plaza.umin.ac.jp/~empower/cec/en/) was designed to fill this gap.

**Figure 1.**
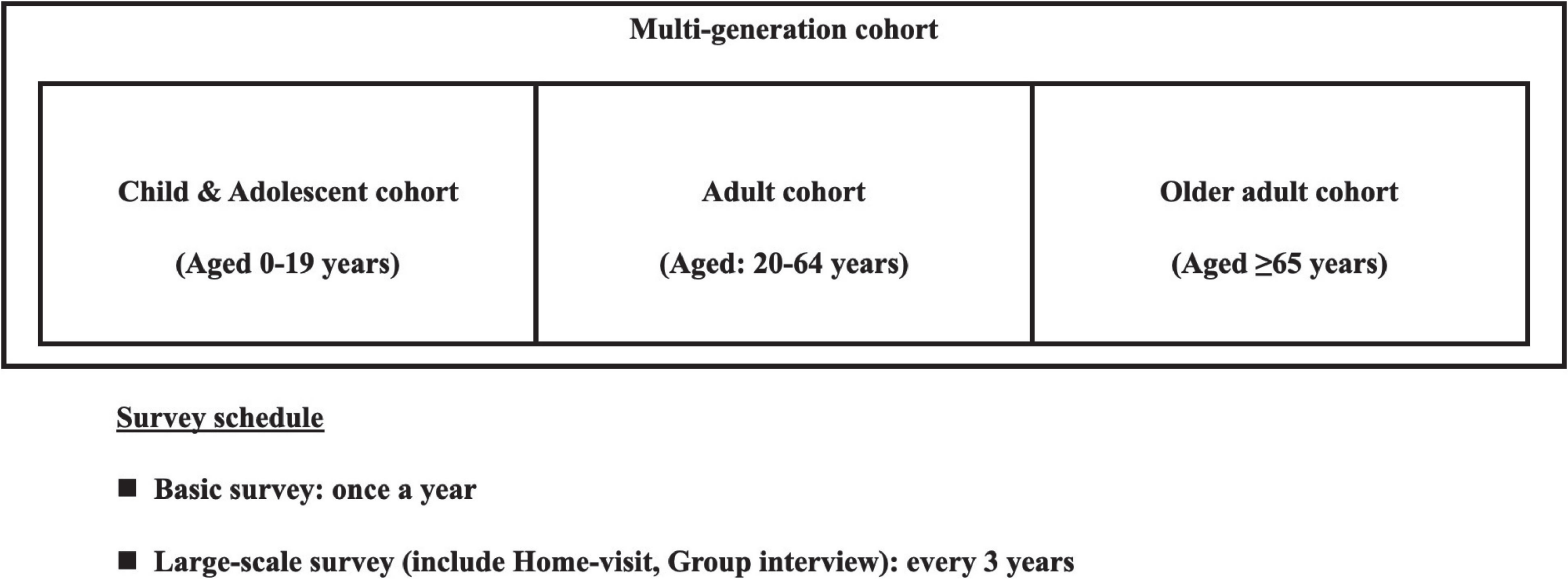
Overview of the Community Empowerment and Care for Well-being and Healthy Longevity (CEC) Study

The CEC Study is a multi-generational cohort involving all residents of Village T and emphasizes community-driven health initiatives (Figure 1). The CEC Study involved participants of all age groups and aimed to empirically validate a life-course approach based on lifespan developmental care.

This community empowerment model encourages participants to engage as co-designers and implementers of health-promotion activities, rather than merely being recipients of services. Residents participate in planning, monitoring, and evaluating local health programs through regular workshops, co-planning meetings, and public feedback sessions. These processes strengthen mutual trust and facilitate evidence-based program refinement. Overall, this approach aims to enhance community well-being by supporting the planning, implementation, and evaluation of scientifically grounded health interventions. Specifically, workshops, focus group interviews, and participatory planning allow residents’ voices to be reflected in health programs, ensuring continuous improvement of intervention strategies. Through this resident-driven approach, the CEC Study goes beyond a traditional health survey to establish a new framework for comprehensive community health promotion.

Furthermore, for more than 30 years, the village has pursued the “Japan’s Healthiest Longevity Village” initiative, a collaborative effort involving local government, residents, and professionals. Various public health programs targeting all age groups, from infants to older adults, have been implemented, with a focus on community empowerment and enhanced well-being. The municipality is anonymized as ‘Village T’ to comply with ethical and data-use requirements aimed at preventing identification of residents, even though the location may be partially inferable. Therefore, Village T functions as an ideal research platform for evaluating long-term health outcomes and generating findings applicable to regions and countries all over the world. The study was initiated in 1991 and has since been jointly implemented by the municipal government and collaborating academic institutions. A key feature of this study is its emphasis on community-wide empowerment, the assessment of intergenerational health, and social interactions. The primary goal of this study was to scientifically evaluate the impact of “Japan’s Healthiest Longevity Village Initiative” and apply its findings to broader social policies. Specifically, by assessing the physical, mental, and social health of residents, this study aimed to achieve the following three objectives:

1. Assess the impact of the Healthy Longevity Initiative:
  • To evaluate changes in key health outcomes over time, including extended healthy life expectancy, reduced risk of chronic disease, and lower costs of medical and long-term care.
  • To provide quantitative evidence by using health check-up data, medical records, and long-term care service usage.
2. Identify factors affecting health and well-being across life stages:
  • To analyze social determinants of health.^14^
  • To examine physical function, mortality, stress levels, quality of life (QOL), and social connections and identify critical intervention areas.
3. Evaluate implementation and develop evidence-based policies:
  • To evaluate implementation processes and policy levels, including the degree of resident participation, program reach and retention, delivery fidelity, and intensity.
  • To relate these implementation indicators to downstream outcomes, such as healthcare utilization, long-term care benefits, and municipal costs, and to create scalable, resident-driven health promotion models adaptable to other regions.

Thus, the CEC Study integrates a life-course perspective with community empowerment to drive local-level health interventions, while providing evidence directly relevant to global health frameworks, such as the WHO’s Decade of Healthy Ageing (2021–2030)^15^ and the United Nations Sustainable Development Goals^16^ (Goal 3: Good Health and Well-being). By demonstrating the feasibility of resident-led, multi-generational health initiatives, this study offers a transferable model for promoting well-being and healthy longevity worldwide.

## MAIN FEATURES

**Figure 2.**
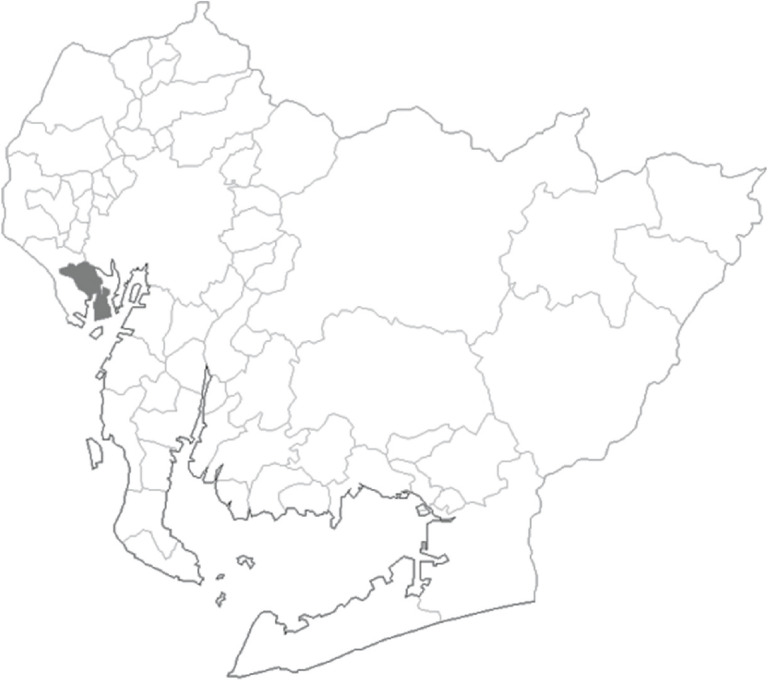
Location of T Village

**Figure 3.**
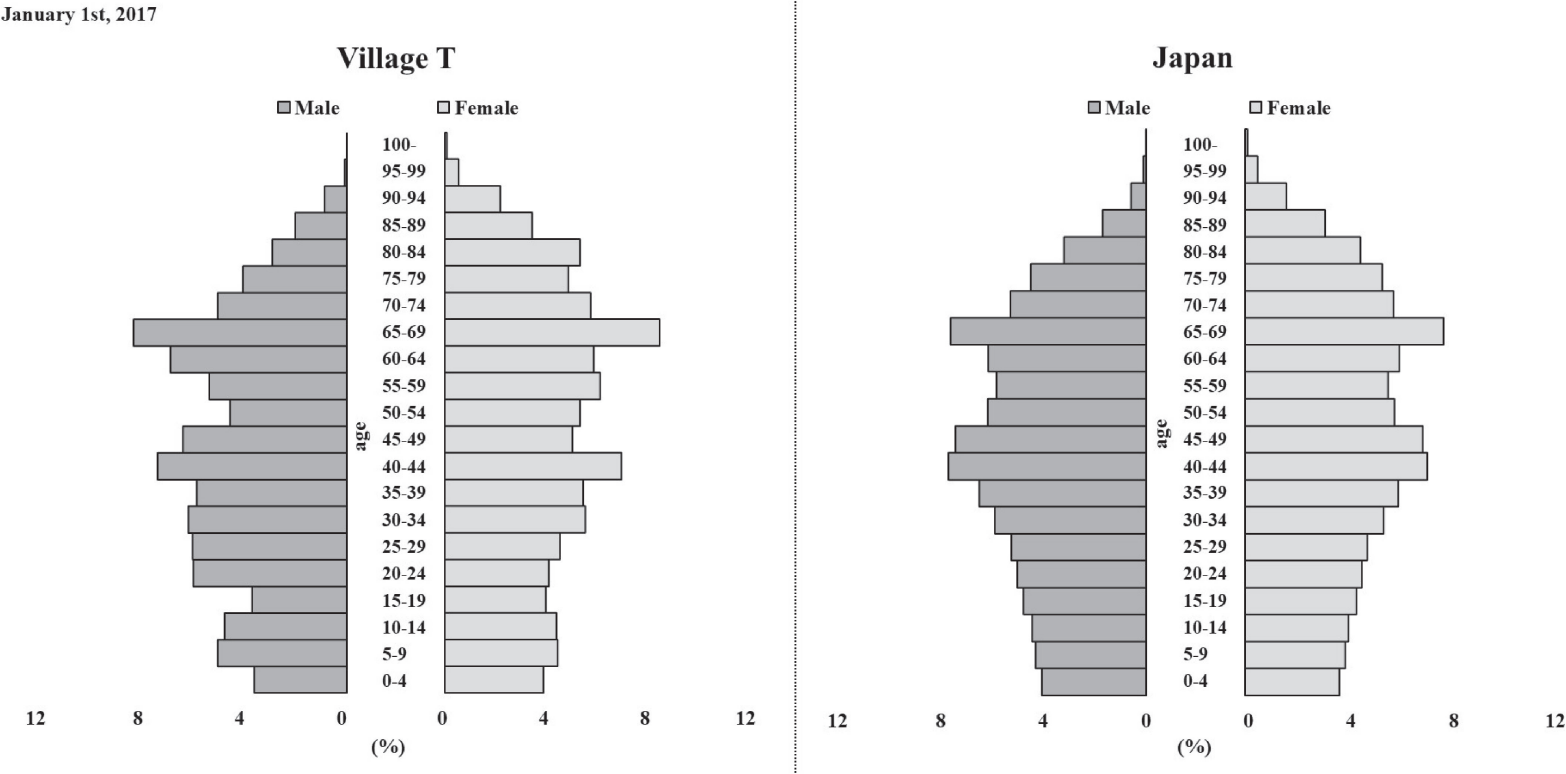
Population pyramid of Japan and T Village in 2017

The Village T is a small municipality located in Aichi Prefecture (Figure 2), with an age distribution that resembles the national population profile. Despite its modest population size, the village shares a broadly similar demographic structure to Japan as a whole, which enables the CEC Study to capture health and social patterns that are relevant in the context of Japan’s rapidly aging society (Table 1 and Figure 3). Table 1 summarizes the basic characteristics of T Village, including population, aging rate, fiscal strength, and proximity to a major metropolitan area. These indicators provide context for interpreting subsequent analyses and clarify the representativeness of the study area. Village T recorded the highest fiscal strength index in 2017 (2.15) in Japan (Ministry of Internal Affairs and Communications). The fiscal strength index is the ratio of a municipality’s revenue-raising capacity to a standard value (1.0 = national standard); 2.15 indicates 215% of the standard capacity. This stable financial foundation of Village T enables the implementation of comprehensive intervention programs for healthy longevity and supports continuous data collection and research.

**Table 1.** Demographic characteristics of Village T and Japan in 2017

	Village T	Japan
Population, *n*	4,615	127,907,086
Young population^a^, *n* (/population) (%)	631 (13.7)	16,142,185 (12.6)
Settlement age population^b^, *n* (%)	2,685 (58.2)	77,491,846 (60.6)
Older adult population^c^, *n* (%)	1,299 (28.1)	34,272,983 (26.8)
Total fertility rate^d^	1.56	1.43
Standardized death rate^e^ (Male/Female)	99.9/107.7	100/100
Persons requiring support of long-term care, *n* (%)	211 (17.1)	6,068,408 (17.9)
• Aged 75 years and older, *n* (%)	182 (24.0)	5,441,398 (32.1)

^a^Young population: aged 0–14 years.

^b^Settlement population: aged 15–64 years.

^c^Older adult population: aged 65 years and older.

^d^Total fertility rate: calculated using the following formula: { $\sum_{15}^{49}$ (number of births by mother’s age/female population by age)}.

^e^Standardized death rate: Calculated using the following formula: {Actual number of deaths/expected number of deaths × 100}.

Furthermore, this study is distinguished by its rich and diverse datasets. Since 1991, approximately 5,000 residents have participated in a total enumeration survey, including questionnaires, home visits, interviews, and health examination data (eg, medical expenses and health check-up records). Since 2017, sub-cohorts have been created, enabling more detailed analyses based on age groups and intervention participation. With this evidence-based design, the CEC Study aims not only to address health issues specific to Village T but also provides universal insights necessary to develop global health policies.

The CEC Study is a multi-generational cohort study involving all residents of Village T, Aichi Prefecture. As of April 2017, Village T had a total population of 4,615, featuring an aging rate of 28.1%, which closely resembles Japan’s national demographic trends. To cover all life stages—from infancy to old age—comprehensively, the study was structured into the following four sub-cohorts.

**Child & Adolescent Cohort (0–19 years)**
  Sample size: Approximately 800 participants
  Focus: To evaluate physical development, lifestyle habits, and educational environment of infants and children.
**Adult Cohort (20–64 years)**
  Sample size: Approximately 2,500 participants.
  Focus: To assess health status, stress levels, and social roles of working-age populations.
**Older Adult Cohort (>65 years)**
  Sample size: Approximately 1,400 participants.
  Focus: To assess frailty prevention, cognitive function, and changes in QOL.

The sample size for each sub-cohort is presented as an approximate number because the cohort frame is defined by the total population in each age group of the municipality, which fluctuates slightly each fiscal year due to births, deaths, and migration. This dynamic framing ensures inclusion of every generation within the community in the cohort base.

## PARTICIPATION

This study employed a total enumeration survey approach involving all residents in Village T. This study was approved by the Ethics Committee of the Morinomiya University of Medical Sciences (approval number 2018-054) and was conducted in accordance with the principles of the Declaration of Helsinki. The requirement for written informed consent was waived, as the analysis used anonymized data, and the participants were allowed to opt out of the study in accordance with the Ethical Guidelines for Medical and Health Research Involving Human Subjects in Japan. Data were collected through questionnaire surveys, home visits, and focus group interviews. Participants were informed about the study by the local government and data were provided in an opt-out format.

The demographic composition of Village T closely mirrors that of Japan, may provide insights that are informative for other regions with similar demographic and socioeconomic profiles. For example, the age distribution among populations in Village T is comparable with Japan’s national census data, and key indicators, including the rate of aging and average life expectancy, are close to the national averages.

Village T is located near Nagoya and represents a regional model for small- and medium-sized cities across Japan. Compared with major metropolitan areas, such as Tokyo and Osaka, Village T may exhibit stronger social cohesion and differences in health behaviors. Future research should explore these regional variations and their implications in public health policies.

## OUTCOMES AND FOLLOW-UP

This study employed a total enumeration survey approach involving all residents in Village T. Data were collected through questionnaire surveys, home visits, and focus group interviews. Residents were informed of the study by the local government, which was responsible for data collection. All the data were anonymized, with personally identifiable information removed prior to transfer. In addition, information pertaining to individuals who had submitted an opt-out request was excluded before the data were shared with the research team.

**Figure 4.**
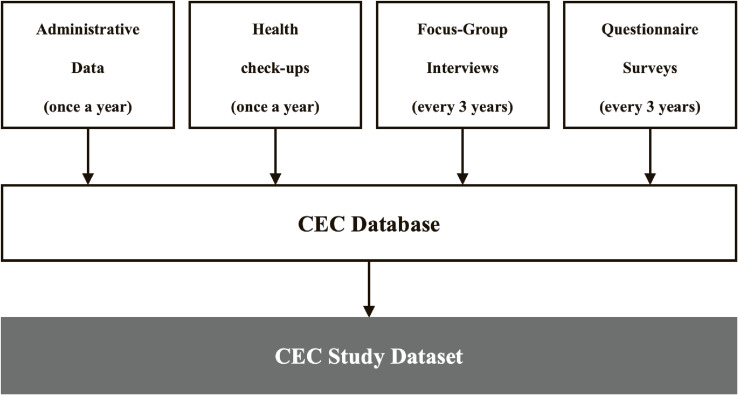
Overview of the integrated Community Empowerment and Care for Well-being and Healthy Longevity (CEC) dataset

In addition, existing administrative data, including health expenditure records and health check-up data, were anonymized and made available for research purposes. By integrating both self-reported and administrative data, comprehensiveness and reliability of the findings in this study were enhanced. An overview of the integrated CEC dataset is presented in Figure 4, which illustrates the data sources and update flow. The dataset includes questionnaire surveys, medical check-up data, home-visit assessments, administrative records, and focus group interviews, enabling comprehensive evaluation of residents’ physical, mental, and social health across all ages.

The CEC Study collected a wide range of data to comprehensively evaluate the overall well-being of participants. As shown in Table 2, the assessments included common evaluation items across both all-age and age-specific groups tailored to unique challenges presented in each life stage. These measurements covered physical, mental, and social health aspects and provided the detailed information necessary to achieve objectives of the study.

**Table 2.** Survey items in each age group

**Child & Adolescent cohort** Aged 0–19 years	**Adult cohort** Aged 20–64 years	**Older adult cohort** Aged ≥65 years
Basic information^a^ ISI^b^ SDQ^c^ Medical check^d^	Basic information^a^ ISI^b^ Medical check^d^	Basic information^a^ Index of Social Interaction^b^ Medical check^d^ Physical function^e^ Long term care certificated status Qualitative data^f^

ISI, Index of Social Interaction; SDQ, Strength and Difficulties Questionnaire.

^a^Basic information: age, sex, standing height, body weight, date of death or emission.

^b^Index of Social Interaction (ISI): independence, social curiosity, interaction, participation in society, feelings of safety as subdomain.

^c^Strength and Difficulties Questionnaire (SDQ): emotional symptoms, conduct problems, hyperactivity/inattention, peer problems, prosocial behavior, total difficulties score as subdomain.

^d^Medical check: blood pressure, waist circumference, red blood cell, hemoglobin, hematocrit, triglyceride, high-density lipoprotein cholesterol, low-density lipoprotein cholesterol, GOT, GPT, γ-GTP, HbA1c, fasting plasma glucose, uric acid, serum creatinine.

^e^Physical function: handgrip strength, single-leg balance with eyes opened, gait speed.

^f^Qualitative data: analysis of raw voices based on focus group interviews.

The index of social interaction (ISI) comprises 18 questions and assesses five subdomains (independence, social curiosity, interaction, participation in society, feelings of safety).^17^ ISI shows highly significant correlations with future health decline and dementia.

The Strengths and Difficulties Questionnaire (SDQ)^18^ is a 25-item survey completed by parents or teachers that assesses children’s emotions and behaviors. It is a screening tool for the overall child mental health, and its reliability and validity have been extensively confirmed. The SDQ is widely used around the world for clinical assessment, school-based screenings, and diverse research purposes.

### Follow-up frequency

Since its inception in 1991, the CEC Study has conducted follow-up surveys every 3 years, systematically tracking health and social factors across all generations. The frequency of follow-ups varied by cohort, incorporating health examination data and survey responses to maintain data accuracy and completeness.

### Follow-up and data update

The CEC database is updated annually based on administrative records provided by the municipal government. Both the self-reported survey data and administrative data are anonymized within the respective administrative agencies and assigned a common unique ID before being provided to the research team. The research team then performs data linkage and analysis using these anonymized IDs as needed.

Individuals newly added to the municipal population, such as those born or moving into the area, are incorporated into the cohort from the following fiscal year’s dataset. This dynamic update process ensures that the database reflects demographic changes and maintains comprehensive coverage of the community population.

### Follow-up schedule

i) Basic survey: basic data of residents were collected annually with the help of the local government, including demographic statistics, the results of regular health checks, data on death, and data on people moving away from the area.
ii) Large-scale survey: a questionnaire survey was conducted every 3 years via mail to collect information on residents’ lifestyles, health conditions, and social relationships.
iii) Home-visit survey: in addition to the large-scale survey, home-visit surveys were conducted to obtain detailed data on subjective health and QOL.
iv) Group interview: focus group interviews were conducted with the target groups, as necessary, to reflect the opinions of residents on policy formulation.

### Follow-up methods for sub-cohorts

Since 2017, follow-up surveys for sub-cohorts have been conducted in addition to overall population surveys. Each sub-cohort underwent the following specific data collection methods:

a) Child Cohort: age-appropriate physical measurements and developmental assessments (eg, physical fitness tests and school health checkups) were conducted.
b) Adult Cohort: in addition to health check-up data, a triennial questionnaire survey was administered to track changes in stress levels, social connections, and lifestyle habits.
c) Older Adult Cohort: to assess frailty prevention and cognitive function changes, a follow-up survey was conducted annually based on the results of health check-up.

### Follow-up objectives and methods

The primary objective of the follow-up surveys was to evaluate mid- to long-term effects of the Healthy Longevity Initiative and utilize the findings to improve existing programs and propose new interventions. To enhance the rate of participation, several measures, including reminder notifications via mail and individual visits, were implemented.

### Cohort retention

To enhance the data collection process and participant retention, individual follow-up visits for non-respondents were conducted whenever possible. To ensure high participation and long-term engagement, the research team and local government maintained bidirectional communication through community health events, feedback newsletters, and participatory review meetings, reinforcing residents’ sense of ownership in the study. Additionally, research findings were shared via municipal publications, such as community calendars and other public media, providing participants with tangible insights on the impact of this study. By fostering a sense of engagement and demonstrating the value of participation, this study encouraged residents to play an active role in the research.

## BASELINE CHARACTERISTICS

This study examined the characteristics of physical and psychosocial indicators by age group. The other parameters are shown in Table 3. In the administrative data, attrition occurs only due to out-migration or death, and no participants have voluntarily withdrawn from the study. For questionnaire surveys, non-respondents are regarded as having withdrawn, and response rates for each survey cycle are presented in Table 3.

**Table 3.** Baseline characteristics

	00–05 years	06–14 years	15–19 years	20–29 years	30–39 years	40–49 years	50–64 years	65–74 years	75–79 years	80–84 years	≥85 years
**Basic information**											
Age, years	2.6	9.8	17.0	24.7	35.0	44.2	57.6	69.1	76.8	81.8	88.9
	(1.66)	(2.49)	(1.44)	(2.97)	(2.89)	(2.73)	(4.24)	(2.76)	(1.37)	(1.4)	(3.32)
male, *n*	88	163	74	136	186	243	331	293	89	65	52
female, *n*	97	158	70	127	188	232	364	314	97	105	94
Standing height, cm	93	138	163	166	165	165	163	158	154	151	149
	(14.7)	(17.7)	(8.2)	(9.1)	(8.5)	(8.6)	(8.4)	(9.04)	(8.9)	(8.5)	(8.8)
Body weight, kg	14.2	34.4	56.3	61	61.8	64.5	61.5	57.9	54.9	53	51.3
	(5.3)	(12.4)	(9.5)	(14.6)	(13.6)	(14.3)	(11.5)	(9.9)	(10.3)	(9.1)	(9.8)

**ISI**^a^ (response rate, %)	(82.2)	(79.7)	(73.8)	(54.0)	(66.8)	(76.2)	(85.7)	(89.7)	(88.6)	(86.3)	(67.0)
Total score	—	15.3	14.8	14.9	15.6	16	16.4	16.4	16.1	15.6	13.7
		(1.7)	(2.2)	(2.2)	(2)	(1.9)	(1.9)	(2.1)	(2.6)	(2.7)	(3.5)
Independence	—	3.69	3.77	3.72	3.74	3.83	3.89	3.9	3.82	3.77	3.55
		(0.68)	(0.75)	(0.72)	(0.71)	(0.58)	(0.44)	(0.45)	(0.6)	(0.63)	(0.83)
Social curiosity	—	3.64	3.8	3.94	4.07	4.28	4.47	4.34	4.02	3.56	2.69
		(0.77)	(0.88)	(0.96)	(0.95)	(0.91)	(0.87)	(1.03)	(1.32)	(1.46)	(1.73)
Interaction	—	2.85	2.72	2.81	2.84	2.83	2.85	2.86	2.77	2.85	2.73
		(0.37)	(0.53)	(0.47)	(0.41)	(0.43)	(0.44)	(0.44)	(0.61)	(0.44)	(0.59)
Participation	—	3.15	2.64	2.51	3.01	3.21	3.31	3.48	3.48	3.48	2.85
		(0.75)	(0.74)	(0.9)	(0.78)	(0.66)	(0.64)	(0.71)	(0.79)	(0.82)	(1.14)
Feelings of safety	—	1.97	1.89	1.94	1.91	1.91	1.87	1.85	1.89	1.88	1.89
		(0.18)	(0.42)	(0.32)	(0.38)	(0.38)	(0.45)	(0.47)	(0.41)	(0.41)	(0.39)

**SDQ**^b^ (response rate, %)		(79.7)									
TDS	—	9.4	—	—	—	—	—	—	—	—	—
		(5.4)									
Prosocial behavior	—	6.5	—	—	—	—	—	—	—	—	—
		(2.3)									
Hyperactivity/inattention	—	3.4	—	—	—	—	—	—	—	—	—
		(2.4)									
Emotional symptoms	—	1.8	—	—	—	—	—	—	—	—	—
		(1.9)									
Conduct problems	—	2.2	—	—	—	—	—	—	—	—	—
		(1.7)									
Peer problems	—	2.1	—	—	—	—	—	—	—	—	—
		(1.7)									

**Medical check ** **(response rate, %)**		12 years old (83.3)	15 years old (91.9)	(1.9)	(20.2)	(29.7)	(34.3)	(47.9)	(52.9)	(52.4)	(26.6)
Systolic blood pressure, Torr	—	—	—	107	107	112	119	126	132	133	134
				(8.4)	(10.8)	(14.6)	(16)	(15.5)	(15.3)	(13.9)	(16.7)
Diastolic blood pressure, Torr	—	—	—	62	64.5	69.6	71.1	72	70.8	71.1	68.9
				(7.3)	(8.3)	(11)	(10.3)	(9.6)	(9.2)	(8.6)	(8.2)
Waist circumference, cm	—	—	—	74.6	79.2	81.3	83.2	83.5	81.7	83.5	84.7
				(5.3)	(9.8)	(10.4)	(9.5)	(8.3)	(9.3)	(9.0)	(5.2)
RBC, 10^4^/µL	—	487	481	470	464	466	462	450	430	417	405
		(30.0)	(39.8)	(47.4)	(45.9)	(45.6)	(39.8)	(44.8)	(43.3)	(43.9)	(48.3)
Hb, g/dL	—	—	—	13.4	13.4	13.6	14	13.7	13.2	12.7	12.4
				(1.9)	(1.7)	(1.8)	(1.3)	(1.3)	(1.3)	(1.4)	(1.3)
Hct, %	—	—	—	41.5	42.1	42.5	43.2	42.5	40.5	39.2	38
				(4.3)	(4.6)	(4.7)	(3.5)	(3.9)	(4.1)	(4.4)	(4.2)
Trigycerides, mg/dL	—	85.7	59.0	49.3	73.6	93.3	113	107	116	122	120
		(41.3)	(36.5)	(10.8)	(46)	(65.9)	(76.4)	(49.4)	(53.2)	(59.4)	(50)
HDL-C, mg/dL	—	65.5	67.1	61.2	65.5	63.6	64.1	61.7	58.6	56.8	52.2
		(16.6)	(14.2)	(12)	(14.6)	(13.4)	(16.1)	(15.5)	(13.8)	(14.4)	(13.5)
LDL-C, mg/dL	—	95.7	94.0	95.2	107	119	124	118	112	112	104
		(25.0)	(22.4)	(13.8)	(24)	(31.8)	(28.5)	(27.9)	(25.5)	(28.6)	(27.9)
GOT, U/L	—	—	—	16.8	20.1	20.9	23.6	24.3	25.1	23.1	24.8
				(3.9)	(7.7)	(11.4)	(12.5)	(8.9)	(8.5)	(6.6)	(6.4)
GPT, U/L	—	—	—	10.8	18.8	20.2	21.6	19.6	17.7	15	14.5
				(3.4)	(15.6)	(17.6)	(18.6)	(12.3)	(7.4)	(6)	(4.8)
γ-GTP, U/L	—	—	—	13.2	31.9	27.4	35.3	33.4	31.3	24.5	21.3
				(3.3)	(104)	(21)	(39.6)	(47.4)	(29.1)	(18.4)	(10.5)
HbA1c, %	—	5.3	5.4	5.3	5.5	5.6	5.9	6	5.9	5.8	5.9
		(0.2)	(0.5)	(0.1)	(0.3)	(0.3)	(0.8)	(0.7)	(0.6)	(0.7)	(0.6)
FPG, mg/dL	—	—	—	82.5	84.3	86.8	91.1	79.5	49.7	48.8	57.2
				(7.9)	(11.2)	(13.5)	(31.8)	(45.4)	(51.6)	(61.8)	(59.4)
uric acid, mg/dL	—	—	—	4.3	4.7	4.8	5.2	5.1	5.2	5.4	5.5
				(1)	(1.4)	(1.4)	(1.2)	(1.3)	(1.4)	(1.4)	(1.5)
serum creatinine, mg/dL	—	—	—	0.7	0.69	0.72	0.74	0.77	0.84	0.82	0.94
				(0.13)	(0.12)	(0.15)	(0.15)	(0.18)	(0.64)	(0.24)	(0.42)

**Physical function ** **(response rate, %)**							(1.1)	(25.3)	(38.6)	(42.1)	(19.7)
Handgrip, kgf	—	—	—	—	—	—	30.8	28.0	26.2	23.7	20.8
							(6.3)	(8.2)	(7.6)	(6.7)	(6.9)
single leg balance, s^c^	—	—	—	—	—	—	11.8	13.4	11.7	9.5	7.4
							(3.4)	(5.3)	(5.8)	(5.8)	(5.0)
gait speed (comfortable), s^d^							3.1	3.4	3.7	4.2	4.4
							(0.4)	(0.5)	(0.7)	(1.1)	(0.9)
gait speed (maximum), s^d^	—	—	—	—	—	—	2.4	2.6	2.9	3.3	3.7
							(0.3)	(0.4)	(0.6)	(1.0)	(1.3)

RBC, red blood cell; Hb, hemoglobin; Hct, hematocrit; ISI, Index of Social Interaction; FPG, fasting plasma glucose; HDL-C, high-density lipoprotein cholesterol; LDL-C, low-density lipoprotein cholesterol; SDQ, Strength and Difficulties Questionnaire; TDS, total difficulties score.

Values are presented as mean (standard deviation).

^a^Index of Social Interaction (ISI): Independence, Social curiosity, Interaction, Participation in society, Feelings of safety as subdomain.

^b^Strength and Difficulties Questionnaire (SDQ): emotional symptoms, conduct problems, hyperactivity/inattention, peer problems, prosocial behavior, total difficulties score (TDS) as subdomain.

TDS (total difficulties score) as subdomain.

^c^Single leg balance: one-legged stance with eyes open.

^d^Gait speed: time required to walk 5 meters.

Participants ranged from 0 to ≥85 years, covering all life stages. Compared with national statistics, participants in Village T exhibited a slightly higher proportion of older adults (28.1% vs 26.8%) and a lower proportion of those aged 0–14 years (13.7% vs 12.6%). Mean body mass index and prevalence of chronic disease were generally similar to national averages.^19^^,^^20^ The ISI score was assessed as a measure of community connectedness across all generations. Because ISI reflects local social structure and contextual factors,^17^ national comparison is not directly applicable; however, its inclusion for all age groups is a distinctive feature of the CEC Study, allowing the evaluation of social participation and mutual support throughout the life course.

## STRENGTHS AND LIMITATIONS

The strengths and limitations of the CEC Study correspond directly to its three primary aims.

1) **Comprehensive coverage across all ages**
  The CEC Study is distinguished by its all-generation design, encompassing participants from infancy to old age. This life-course coverage allows longitudinal tracking of physical, mental, and social health trajectories and supports both long-term panel and generation-specific analyses. In comparison with other major cohorts such as UK Biobank,^21^ National Health and Nutrition Examination Survey,^22^ and Japan Gerontological Evaluation Study,^23^ the CEC provides a uniquely comprehensive perspective on community health across the lifespan.
2) **Identification of life-course determinants**
  By integrating questionnaires, health-check data, home-visit assessments, and interviews, the study enables examination of biological, psychological, and social determinants that influence health and well-being. Linkage with medical and long-term care records further supports the analysis of life-course determinants of healthy longevity, yielding evidence applicable to national and local policy development.
3) **Resident participation and implementation evaluation**
  The CEC emphasizes community empowerment by evaluating implementation processes at both community and policy levels. Active collaboration among local government, residents, and professionals ensures feasibility of long-term follow-up and allows measurement of implementation indicators such as participation, reach, retention, fidelity, and continuous quality improvement processes (eg, annual program review and refinement). This design directly links research findings with continuous policy feedback and program improvement.

### Limitations

Several limitations should also be noted. First, because the CEC is conducted in a single municipality with relatively high fiscal capacity, the generalizability of findings to less affluent or structurally different regions may be limited. Second, the modest population size (∼4,600) reduces statistical power for subgroup analyses. Third, self-reported measures may involve recall or social-desirability bias, and discrepancies between administrative and survey timing could introduce measurement error. Nevertheless, the CEC Study provides a unique long-term, community-embedded framework for evaluating multi-generational health promotion and empowerment strategies.
